# Cholecystectomy and common bile duct exploration via a ventral hernia sac. A novel solution for a co‐morbid patient

**DOI:** 10.1111/ans.19262

**Published:** 2024-11-11

**Authors:** Mira Prashar, Jai Hoff, Kellee Slater

**Affiliations:** ^1^ Faculty of Health Sciences and Medicine Bond University Gold Coast Queensland Australia; ^2^ Department of General Surgery Princess Alexandra Hospital Brisbane Queensland Australia

A 69‐year‐old female was referred to the Abdominal Wall Reconstruction Unit with a ventral hernia and five episodes of gallstone pancreatitis. The patient's hernia had persisted since her child‐bearing years and had undergone four unsuccessful repairs. Her current hernia demonstrated a 95% loss of domain M1, W3 configuration and had been stable for 10 years. Almost all of the large bowel, small bowel, right kidney, right lobe of the liver and gallbladder were contained in the hernia sac, extending into a Grade 3 abdominal apron. She was a diabetic and had undergone sleeve gastrectomy for weight loss 10 years prior. Current body mass index was 38 kg/m^2^ but had been maximal at 60 kg/m^2^. The patient had limited mobility, using a wheelchair given her body habitus and osteoarthritis. It was our opinion that her ventral hernia was inoperable, with serious mortality risk.

Despite these frailties, the patient enjoyed her social life and wished to be relieved of her episodes of gallstone pancreatitis. Each attack resulted in pain, jaundice and cholangitis with lengthy hospitalisations.

Computed Tomography revealed cholelithiasis and the gallbladder superficially positioned in the hernia sac (Fig. 1). Magnetic Resonance Cholangiopancreatography confirmed choledocholithiasis, with five gallstones in the common bile duct. Laparoscopic cholecystectomy and common bile duct exploration were deemed technically impossible due to the ventral hernia. The patient was referred for Endoscopic Retrograde Cholangiopancreatography to attempt to treat the choledocholithiasis and whilst not addressing the stones in the gallbladder, may have been sufficient to reduce the attacks of pancreatitis and cholangitis. This was unsuccessful due to the duodenal anatomy being distorted by the eviscerated hernia and a large duodenal diverticulum.

**Fig. 1:**
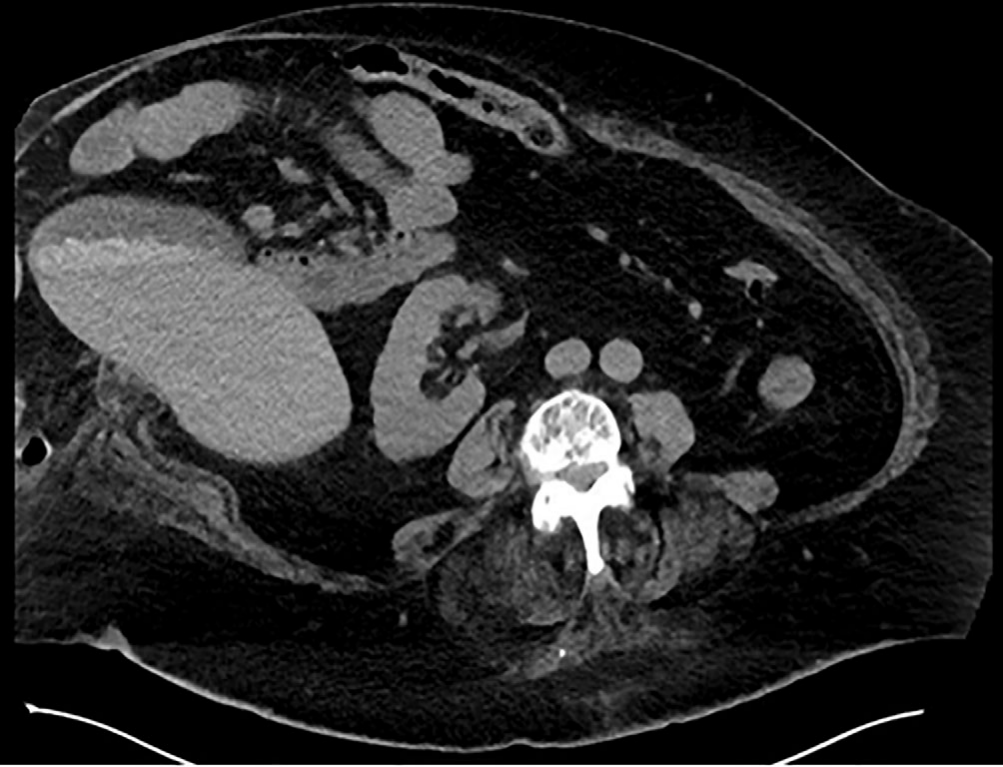
Extension of the right hemiliver and gallbladder containing numerous stones outside the abdominal musculature due to an M1, W3 ventral hernia.

As the hernia had rotated the gallbladder into an anterior, superficial position, a less conventional approach was considered. We performed an open cholecystectomy and transcystic bile duct exploration through the hernia sac.

Under general anaesthesia, the patient's gallbladder position within the hernia sac was confirmed using transcutaneous ultrasound. A 5‐cm transverse incision was made over the gallbladder in a trajectory that offset tension on the skin wound (Fig. 2). The hernia sac was opened and a small Alexis retractor (Applied Medical) was deployed into the wound and sac (Fig. 3). The gallbladder was easily accessible and retrograde cholecystectomy was performed. Transcystic choledochoscopy confirmed numerous stones in the common bile duct, which a basket retrieved. An intraoperative cholangiogram confirmed clearance.

**Fig. 2:**
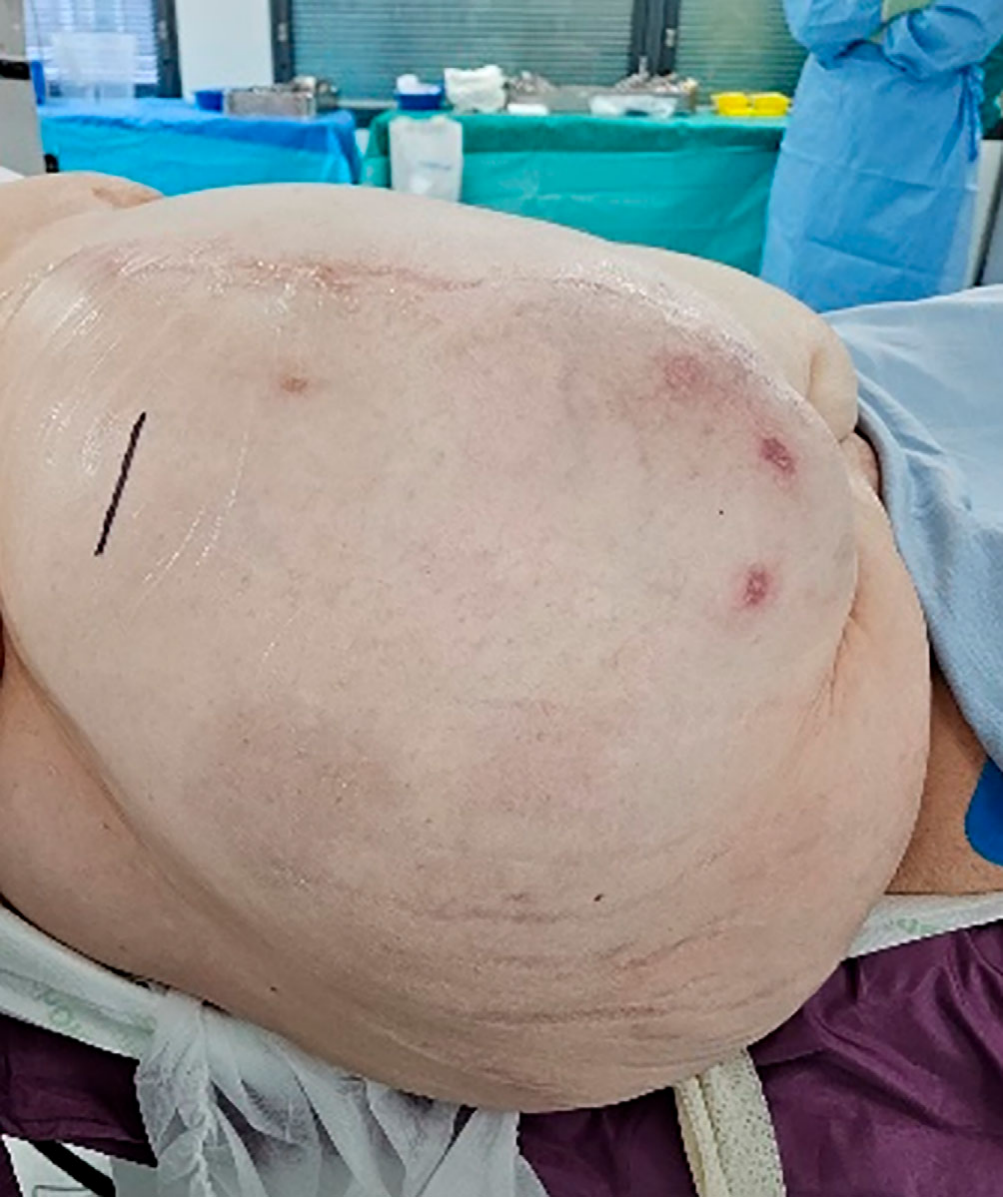
Ultrasound guided confirmation of the gallbladder in the hernia sac, prior to the transverse incision.

**Fig. 3:**
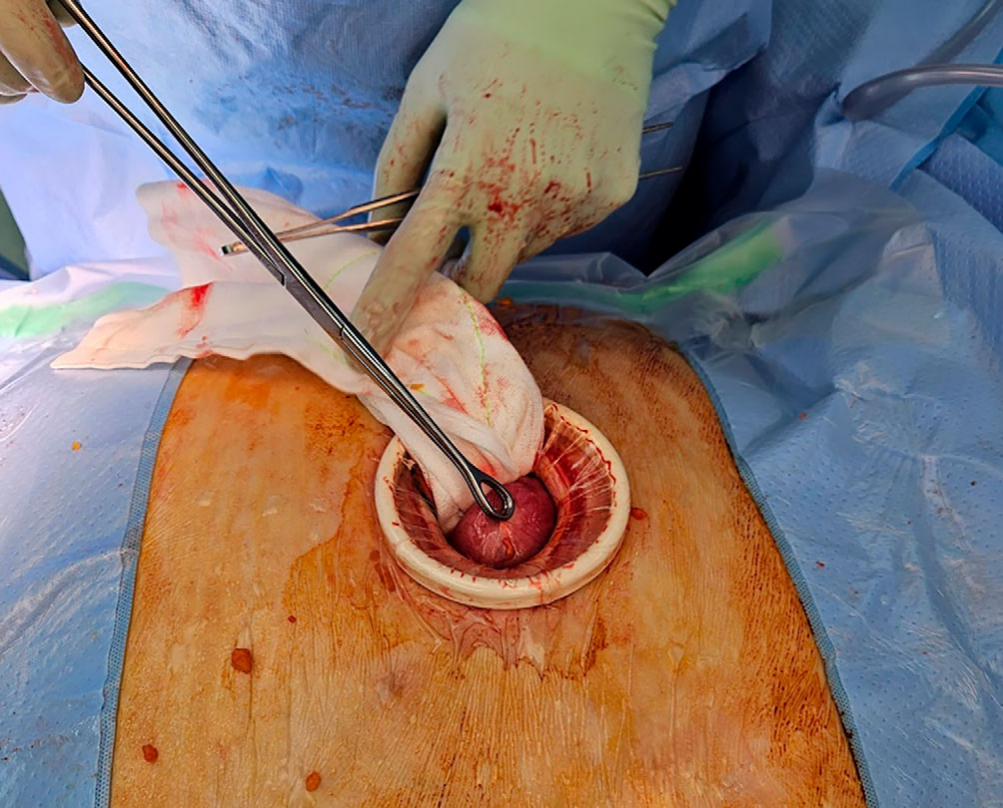
The gallbladder's proximity to the abdominal wall, makes it simple to access through the skin and hernia sac.

The sac, subcutaneous fat and skin were closed with absorbable suture and a Prevena (3M) vac was placed over the wound for 5 days. The patient had minimal discomfort and was discharged on day five following dressing removal. The wound healed non‐incidentally.

A literature review was conducted and no similar cases have been reported. Due to advances in medical technology, patients are living longer with significant co‐morbidities. Australia's obesity epidemic presents General Surgeons with medical problems impacting quality of life that may not be amenable to traditional management. Imaging and device technology allow us to employ innovative solutions and achieve good outcomes for patients. This case demonstrates one such approach.

## Author contributions

**Mira Prashar:** Writing – original draft; writing – review and editing. **Jai Hoff:** Writing – review and editing. **Kellee Slater:** Writing – original draft; writing – review and editing.

